# Hepatitis B Vaccine Nonresponse and Associated Risk Factors: Insights From a Cohort Study

**DOI:** 10.1155/jotm/3879562

**Published:** 2025-03-27

**Authors:** Shuaibu Abdullahi Hudu, Abdulgafar Olayiwola Jimoh, Sa'adatu Haruna Shinkafi, Albashir Tahir, Ahmed Subeh Alshrari, Muhammad Tukur Umar, Abdulmajeed Yunusa, Nura Bello, Nura Abubakar

**Affiliations:** ^1^Department of Basic and Clinical Medical Sciences, Faculty of Dentistry, Zarqa University, Zarqa 13110, Jordan; ^2^Department of Microbiology and Parasitology, Faculty of Basic Clinical Sciences, College of Health Sciences, Usmanu Danfodiyo University, Sokoto 840232, Nigeria; ^3^Department of Pharmacology and Therapeutics, Faculty of Basic Clinical Sciences, College of Health Sciences, Usmanu Danfodiyo University, Sokoto 840001, Nigeria; ^4^Department of Microbiology and Parasitology, Usmanu Danfodiyo University Teaching Hospital, Sokoto 23270, Nigeria; ^5^Department of Pharmacology, Faculty of Basic Medical Sciences, Bauchi State University, Gadau 751105, Nigeria; ^6^Medical Laboratory Technology Department, Faculty of Applied Medical Science, Northern Border University, Arar 91431, Saudi Arabia; ^7^Department of Pharmacology and Therapeutics, Faculty of Pharmaceutical Sciences, Ahmadu Bello University, Zaria 810107, Kaduna, Nigeria; ^8^Department of Physiotherapy, Faculty of Basic Health Sciences, Zamfara State University, Talata Mafara 892001, Nigeria

**Keywords:** hepatitis B virus, immune response, nonresponders, risk factors, vaccination

## Abstract

Hepatitis B virus (HBV) infection remains a critical global health issue, particularly in high-burden regions with substantial global morbidity and mortality. Despite the efficacy of the hepatitis B vaccine in inducing immunity, a subset of vaccinated individuals fails to achieve adequate immunity. This study investigated the factors influencing immunological response to the hepatitis B vaccine among Nigerian adults. A cohort study was conducted at Usmanu Danfodiyo University Teaching Hospital, Sokoto, involving 307 participants aged 19–60 who had not previously received the hepatitis B vaccine. Data were collected using structured questionnaires assessing demographic characteristics, medical history, and lifestyle factors. Participants received GeneVac-B, a recombinant hepatitis B vaccine, and 0-, 1-, and 2-month schedule anti-HB levels were measured post-vaccination using a commercial enzyme-linked immunosorbent assay (ELISA) kit (AccuDiag ELISA, Diagnostic Automation/Cortez Diagnostics, Inc., USA) to classify vaccine response. The association between demographic and lifestyle factors and vaccine response was analyzed using IBM SPSS Statistics (Version 25). Of the 307 participants, 209 received the first vaccine dose, and 192 completed all three doses. Anti-HB quantification revealed that 94.3% of the participants achieved protective immunity (≥ 10 IU/L), while 5.7% were classified as nonresponders (< 10 IU/L). Statistical analysis indicated significant differences in the immune response based on gender (*p* = 0.031), with females exhibiting higher mean anti-HB levels than males. Negative correlations were observed between age and vaccine response (*ρ* = −0.296, *p* < 0.01) and between body mass index (BMI) and response, although the latter was not statistically significant (*ρ* = −0.131, *p* = 0.071). Prior tuberculosis, malaria, measles, smoking, and alcohol use showed no significant impact on the immune response, although recurrent malaria, measles, and smoking were associated with slightly lower mean antibody levels. The study indicates gender and age significantly influence hepatitis B vaccine response, with females and younger individuals demonstrating stronger immunity.

## 1. Introduction

The hepatitis B virus (HBV) infection continues to be a pressing health challenge worldwide, with significant morbidity and mortality associated with chronic liver disease, cirrhosis, and carcinoma of the liver. HBV is a DNA virus belonging to the Hepadnaviridae family, primarily targeting hepatocytes and resulting to both acute and chronic infections. The World Health Organization (WHO) estimated that about 254 million persons worldwide suffered from chronic HBV infection, with an annual incidence of 1.2 million new cases and around 1.1 million deaths linked to HBV-related complications, predominantly in high-burden regions such as the Western Pacific and Africa [1]. Inadequate vaccination coverage, restricted access to healthcare facilities, cultural practices, and vertical transmission during childbirth contribute to the persistent dissemination of HBV infection [2, 3]. In Nigeria, HBV remains a significant public health concern, with a national seroprevalence of about 8%, categorizing the country as hyperendemic [4]. The prevalence varies across different populations, with higher rates observed in healthcare workers, blood donors, pregnant women, and individuals with high-risk behaviors [4]. Despite the introduction of routine infant immunization and targeted vaccination campaigns for adults, challenges persist in achieving widespread immunity due to vaccine nonresponse and poor vaccine uptake [5]. Understanding the factors influencing HBV vaccine response is crucial in Nigeria, where gaps in immunization coverage and disease control strategies still exist.

The predominant HBV genotype in Nigeria and West Africa is genotype E, with epidemiological studies consistently reporting its dominance. In Southeastern Nigeria, genotype E accounts for 75% of infections, followed by genotype A (10%) and mixed A/E infections (15%) [6]. This aligns with findings from other regions: Jos, Nigeria, showed exclusive genotype E prevalence among chronic HBV patients [7], while studies in Benin City, Port Harcourt, and Zaria (Nigeria) similarly identified genotype E as the most prevalent [6]. Across West Africa, genotype E is endemic and rarely detected outside the continent, suggesting a recent evolutionary origin localized to this region [6, 8].

The primary intervention for HBV control is vaccination, which has demonstrated significant efficacy in preventing infection and reducing disease burden, particularly when administered universally in highly endemic regions. The hepatitis B vaccination triggers the production of antibodies against the virus, providing long-term immunity [9–11]. This vaccine has been pivotal in reducing the incidence of chronic liver illnesses and is one of the first vaccines to prevent cancer by reducing the risk of hepatocellular carcinoma [12]. However, a subset of vaccinated individuals, estimated at 5%–10%, fails to develop sufficient antibody levels for protection, even after completing the recommended vaccination series. These individuals, classified as “nonresponders,” represent a challenge to HBV control efforts as they remain susceptible to infection despite vaccination [13]. The variability in immunological response to the hepatitis B vaccine has been attributed to several factors including an individual's age, gender, body mass index (BMI), immune status, and genetic predispositions [13, 14]. For instance, immunosenescence, a decline in immune function with age, has been linked to reduced responses to many vaccines, including hepatitis B. Additionally, gender differences in immune responses are well documented, with females typically mounting stronger responses than males. Obesity and underlying medical conditions, such as chronic infections or immune disorders, also affect the vaccine's efficacy [13]. Biological factors such as high cholesterol levels, high triglycerides [15], and the microbiota also influence the degree of seroprotection achieved [16].

This is a significant challenge for nonresponders within high-risk groups, including healthcare professionals, individuals residing in endemic areas, and individuals engaging in unprotected sexual activities with multiple partners or using intravenous drugs. Several studies conducted in Africa have provided valuable insights into hepatitis B vaccine response among different populations. A study in Ghana by Vivian Efua, Armah, and Delali Adwoa assessed the serological response to the hepatitis B vaccine among healthcare workers and found that a significant proportion (91.3%) achieved protective antibody levels post-vaccination [17]. Similarly, a cross-sectional study in Cameroon by Anutebeh et al. examined immune responses in fully immunized children and highlighted variations in seroprotection based on age and other demographic factors [18]. In Uganda, Ocan et al. investigated antibody levels among adult healthcare workers and reported varying degrees of seroprotection, emphasizing the need for booster doses in certain individuals [19]. While several studies have explored factors influencing vaccine response, similar research in the Nigerian context is scarce, as existing studies have primarily focused on epidemiology [4, 20]. This situation can perpetuate transmission within communities, undermining public health efforts aimed at controlling HBV infections. The lack of such studies within the Nigerian population underscores the need for further research in this area to develop tailored vaccination strategies. Thus, this study aimed to examine the factors that could influence the immune response to the hepatitis B vaccine. This study examines the immunological response to the GeneVac-B hepatitis B vaccine among at-risk Nigerian adults, identifying factors contributing to vaccine nonresponse.

## 2. Methods

### 2.1. Study Design, Setting, and Population

This study utilized a cohort study design, involving individuals of the Nigerian indigene, residing in Sokoto State who consented to participate, and of all demographic characteristics. The study was conducted at the Infectious Diseases Laboratory of Usmanu Danfodiyo University Teaching Hospital, Sokoto, targeting individuals who had previously received the hepatitis B vaccine. The inclusion criteria were participants aged 19–60 years at enrollment, with no prior history of hepatitis B vaccination, and no known conditions that could affect their participation. Exclusion criteria included participants those who tested positive for hepatitis C.

### 2.2. Sampling Technique

A convenience sampling technique was utilized to select participants based on their willingness to participate and accessibility during the study period. This approach was chosen due to feasibility constraints and accessibility. Diverse representations across age groups, genders, and demographic backgrounds were ensured to include individuals from different backgrounds to minimize bias.

### 2.3. Determination of Sample Size

The sample size was calculated using the sample size formula for proportions [21]:

(1) $$\begin{array}{c} n=\frac{Z^{2}*p*\left(1-p\right)}{E^{2}}, \end{array}$$

where *n* denoted the required sample size, *Z* is the *Z* score corresponding to the desired confidence level (approximately 1.96 for a 95% confidence level), *p* is the estimated prevalence of nonresponse (10% or 0.10) [13], and *E* is the margin of error (set at 5% or 0.05). Given these parameters, the sample size was calculated to be 139. Taking into consideration participants' attrition during the study, the sample size was adjusted for a 10% (0.10) attrition rate, resulting in a final sample size of 151 using the following formula:

(2) $$\begin{array}{c} \text{New Sample Size}=\frac{\text{Original Sample Size}}{1-\text{Attrition Rate}},\\ \text{New Sample Size}=\frac{136}{\left(1-0.10\right)},\\ \text{New Sample Size}=\frac{136}{\left(0.90\right)}\approx 151. \end{array}$$

### 2.4. Data and Sample Collection

Participants' data including age, gender, BMI, smoking status, alcohol use, and comorbidities were collected using a structured questionnaire. HBV and HCV statuses were assessed using qualitative tests. The participants' data were collected through structured questionnaires and clinical assessments (HBV, HCV, height, and weight). The questionnaire was pretested with a small sample, and feedback was used to clarify and refine the items to ensure reliability and comprehensibility. HBV and HCV statuses were assessed using qualitative tests. Trained personnel conducted the data collection, ensuring adherence to standardized protocols. Participants' medical histories and lifestyle factors were also documented to evaluate potential risk factors for vaccine nonresponse.

### 2.5. Vaccine Administration

Participants who met the inclusion criteria received a 20 μg dose (in 1.0 mL suspension) of the hepatitis B vaccine (GeneVac-B) intramuscularly in three doses, following a 0-, 1-, and 2-month accelerated schedule. While the standard hepatitis B vaccination schedule in Nigeria follows a 0-, 1-, and 6-month regimen for adults, accelerated schedules have been used in certain high-risk populations to achieve earlier immunity [22]. Anti-HB levels were quantified to classify response status: participants with anti-HB levels ≥ 10 IU/L were considered responders, whereas those with levels < 10 IU/L were classified as nonresponders. A booster dose with a different brand (Euvax B) was administered to participants who did not develop the required antibody levels from the first initial vaccination using a similar schedule, and the anti-HB titers were measured four weeks after the final vaccine dose to assess immunological response.

### 2.6. Laboratory Analysis

#### 2.6.1. Specimen Collection and Storage

A trained phlebotomist collected about 4-5 mL of venous blood samples in 5 mL ethylenediaminetetraacetic acid (EDTA) bottles following strict aseptic and safety protocols. The samples were labeled using the participants' IDs, centrifuged, and the serum extracted and stored according to standardized protocols for subsequent analyses.

#### 2.6.2. HBV Profile Test

The Hightop HBV 5-in-1 Hepatitis B Virus Marker Rapid Test Panel (Qingdao Hightop Biotech Co. Ltd., China) was used to qualitatively screen HBV markers. Before testing, the test kit was allowed to equilibrate for 30 min. Three drops (80–100 μL) of serum were applied vertically to the sample well (S) and observed for 15–20 min, and the results were interpreted as follows:

  HBsAg, HBsAb, and HBeAg: A red line in the control (C) region, alongside another red line in the test (T) region (even if faint), was interpreted as a positive result. A red line in the C region with no line in the T region indicates a negative result. Results were considered invalid if no red lines appeared or if the control line failed to appear, and the test was repeated for such cases.
  HBeAb and HBcAb: A red line in the C region, with either no line or a faint line in the T region, was interpreted as a positive result, while two distinct red lines observed in both regions indicated a negative result. The absence of red lines or failure of the control line to appear rendered the test invalid, necessitating a repeat test.

#### 2.6.3. Qualitative HCV Test

One Step Hepatitis C Virus Test Strip (Qingdao Hightop Biotech Co. Ltd., China) was used for the qualitative detection of HCV antigens. The test strips were equilibrated at room temperature before testing. About 10 μL of plasma was applied to the test strip using a pipette, followed by the addition of 40 μL of buffer, and the results were recorded after 10 min. The appearance of two separate lines in the control and test regions signified a positive result, whereas a single red line in the control region, without any visible line in the test region, indicated a negative result. If no red line appeared in the control region, the test was considered invalid and was repeated.

#### 2.6.4. Assessment of Anti-HB Titers

A commercial enzyme-linked immunosorbent assay (ELISA) kit (AccuDiag ELISA) using the sandwich ELISA principle was used to quantify anti-HB levels according to the manufacturer's instructions (Diagnostic Automation/Cortez Diagnostics, Inc., USA). Before the tests, the reagents and samples were thawed for 15–30 min. The required strips were placed in a holder, and about 50 μL of calibration curve standards and test samples was added to their respective wells. Fifty microliters of HRP-conjugate reagent was added to each well except for the blank and stirred gently. The plates were covered and incubated for 60 min at 37°C. Following incubation, each well was washed five times with diluted wash buffer and allowed to soak for 30–60 s. Fifty microliters of Chromogen A and B solution was dispensed into each well, mixed by gently tapping the plate, and incubated at 37°C for 15 min. The enzymatic reaction between the Chromogen A/B solutions and the HRP-conjugate produced a blue color in calibration curve standard wells (except for 0 IU/L) and anti-HB-positive sample wells. The reaction was terminated by the addition of 50 μL sulfuric acid to each well, which turned the blue color yellow. After calibrating the plate reader, the absorbance was measured at 450 nm. The results were computed by deducting the blank well's optical density (OD) from the sample and control values.

### 2.7. Data Analysis

Unique identification codes were assigned to the participants, and data were entered into a Microsoft Excel file. The database was exported to the IBM SPSS software for analysis. Descriptive statistics were used to summarize participants' characteristics, medical history, and lifestyle factors using frequencies and percentages. Differences in the means of vaccine response (anti-HB levels) between gender, chronic diseases, tuberculosis (TB), measles, malaria, smoking, and alcoholism were compared using an independent samples *t*-test. The Spearman correlation coefficient was used to determine the association between age, BMI, and vaccine response. SPSS software (Version 25) was used for statistical analyses, and a *p* value of ≤ 0.05 was considered statistically significant.

### 2.8. Ethical Approval

Ethical approval was obtained from the Ethics Committee of the Usmanu Danfodiyo University Teaching Hospital (approval number: UDUS/DRID/BMREC/16062022/25). Written informed consent was obtained from all participants, after ensuring that they were fully informed about the study and that their rights and confidentiality were strictly protected.

## 3. Results

### 3.1. Participants' Sociodemographic Information

A total of 307 participants were recruited for the study, comprising 36% females (*n* = 117) and 64% males (*n* = 190). Following the initial screening, 226 participants met the inclusion criteria. The excluded participants included 20 who tested positive for HBsAg, 42 for anti-HBs, 16 for anti-HBc, and 3 for HCV. Ultimately, 209 participants were successfully administered the first vaccination dose. Of these, 192 completed all three vaccine doses and their anti-HBs were quantified, whereas 32 participants were lost to follow-up. Among the 192 participants included in the final analysis, 116 were males and 92 were females. Demographic information, including age, weight, height, and BMI, is summarized in Table 1.

### 3.2. Medical Histories of Vaccinated Participants

**Figure 1:**
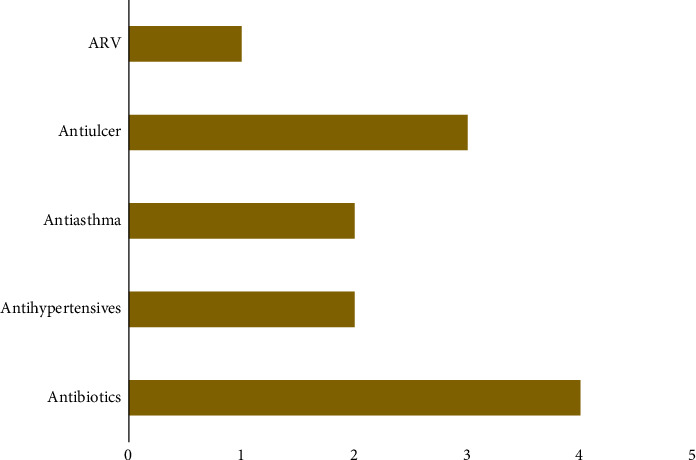
Medication use among participants.

Among the total participants, six individuals reported a previous history of hepatitis B vaccination, while ten participants indicated that they had chronic diseases. Of these, two reported having hypertension, three reported asthma, four had peptic ulcer disease, and one reported HIV status. In addition, 12 participants were currently on medication. Medication usage among the vaccinated participants is depicted in Figure 1.

Distribution of commonly used medications among participants.

### 3.3. History of Infectious Diseases Among Vaccinated Participants

**Figure 2:**
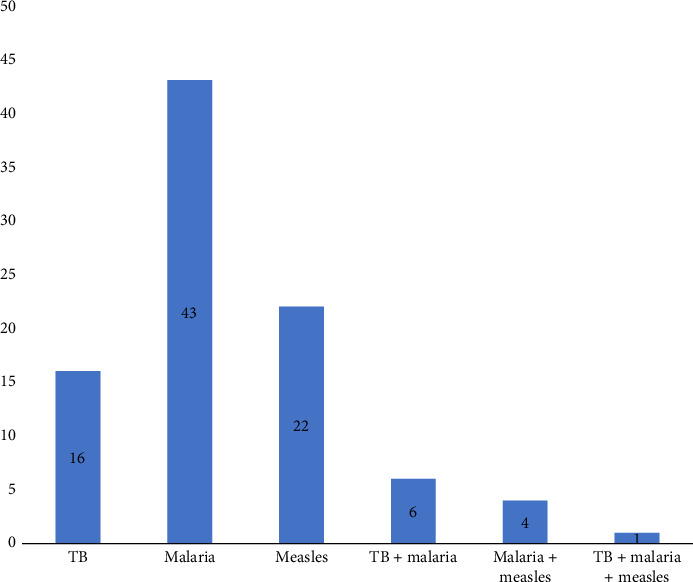
History of infectious diseases.

Participants were asked to report any past occurrences of TB, malaria, measles, or combinations of these diseases; responses are summarized in Figure 2.

Histogram showing the prevalence of TB, malaria, and measles among participants with infectious diseases.

### 3.4. Lifestyle Factors

Regarding smoking and alcohol consumption habits, two participants reported smoking, six indicated that they consumed alcohol, and one reported both smoking and alcohol consumption.

### 3.5. Anti-HB Quantification

Following quantification, 11 participants (5.7%) had anti-HB titers below 10 IU/L, indicating inadequate immunity. In contrast, 181 participants (94.3%) attained an anti-HB level of 10 IU/L or higher, which is considered protective against HBV infection.  Table 2 summarizes the mean responses to the vaccine across different variables.

The results show a statistically significant difference in anti-HB response based on gender, with females exhibiting a higher mean response than males (*p* = 0.031). Anti-HB levels were lower in smokers and those with a history of TB, recurrent malaria, and measles, while alcohol consumers presented a higher anti-HB titer. All these observed differences were not statistically significant.

The majority of nonresponders were male (81.8%, *n* = 9), with only two females (18.2%) failing to develop protective antibody levels. Nonresponders were generally older (mean: 34.8 years) compared to responders (mean: 26.5 years) and had a higher mean BMI (24.1 kg/m^2^) compared to responders (20.3 kg/m^2^). The Spearman correlation coefficient (*ρ*) analysis revealed a moderate negative association between age and vaccine response, indicating that as age increases, the immune response to the vaccine decreases significantly (< 0.01). The correlation between BMI and vaccine response is weak and not statistically significant (*p* = 0.071). However, the *p* value is close to significance, suggesting a potential trend where higher BMI may be associated with lower vaccine response.

## 4. Discussion

This study evaluated the immune response to the GeneVac-B hepatitis B vaccine in a Nigerian adult population and identified factors associated with vaccine nonresponse. The results indicate that 94.3% of participants developed protective immunity (anti-HBs ≥ 10 IU/L), while 5.7% were classified as nonresponders (anti-HBs < 10 IU/L). These findings are consistent with global estimates, where 5%–10% of vaccinated individuals fail to achieve protective antibody levels, highlighting the need to understand nonresponse in specific populations [13]. The immune response to the vaccine was statistically different between genders, with females having a higher mean response than males (*p* = 0.031), which aligns with previous studies that showed gender differences in response to the vaccine [23]. This difference was related to the opposing immunological effects of sex hormones. Testosterone suppresses the production of immunoglobulin G (IgG) and immunoglobulin M (IgM) by B-lymphocytes. Additionally, it suppresses the production of interleukin-6 (IL-6) by monocytes. Estrogen triggers the release of IgG and IgM resulting from its stimulation of IL-10 release from monocytes [23–25].

History of TB and recurrent malaria, as shown by high *p* values (0.970 and 0.362, respectively), did not show significant differences in anti-HB levels (Table 2). The lack of a significant impact of TB and malaria history implies that these conditions may not influence the immunological response to hepatitis B vaccination in the study population. Still, studies have shown that these conditions may affect the vaccine response. For instance, TB infection promotes chronic immune activation and dysregulation, which may impair the host's ability to develop an efficient immune response to vaccines. *Mycobacterium tuberculosis* alters cytokine profiles, resulting in the predominance of anti-inflammatory cytokines that could hinder the development of the essential immune response needed to optimize protection after hepatitis B vaccination [26]. Amplified frequencies of regulatory B cells (Bregs) have been reported in patients with TB. These cells can suppress immune responses by altering the microenvironment of cytokines, which may in turn inhibit the production of antibodies against hepatitis B. Hepatitis B vaccine nonresponders with elevated levels of IL-10 have a higher frequency of CD24^high^CD38^high^ Bregs [26].

The response to the hepatitis B vaccine was lower in participants with recurrent malaria than those without (203.54 ± 135.03 vs. 224.77 ± 133.89). While this difference was not of statistical significance (*p* = 0.362), studies have shown that recurrent malaria can induce immune suppression, which may interfere with the body's ability to mount effective responses to vaccinations. This suppression affects immune cells such as B cells and T helper cells, which are critical for generating an adequate antibody response to vaccines [27, 28]. During malaria infection, particularly with *Plasmodium falciparum*, there is an observed increase in Th2 cytokines such as IL-4 and IL-10 [29]. The predominance of Th2 responses can lead to a state of immune suppression, which may hinder the ability to mount effective responses against other pathogens, including viruses such as HBV. The anti-inflammatory environment created by Th2 cytokines may dampen the overall immune response, potentially reducing the vaccine's efficacy [30].

Participants with a history of measles exhibited a lower mean immunological response to the vaccine than those without such a history (Table 2), although the difference was not statistically significant (*p* = 0.090). The observed difference can be attributed to immune amnesia, a condition in which the body's immune system loses memory of previous infections and vaccinations, including the hepatitis B vaccine, because measles infection damages immune cells (specifically, memory B and T cells) [31, 32]. The measles virus preferentially attacks memory T cells due to its increased expression of the CD150 receptor, which facilitates viral entry. This results in the depletion of memory cells during the acute phase of measles infection. Studies have shown that CD4+ and CD8+ memory T cells are significantly affected, reducing their population post-infection [33]. Similarly, research indicates that not only are pre-existing memory B cells destroyed, but the overall repertoire of B cells is also diminished, reducing their ability to respond to a variety of pathogens [34].

The impact of smoking on anti-HB levels in the study population was minimal, with both smokers and nonsmokers showing comparable responses and a high *p* value (0.464). Notably, individuals who consumed alcohol demonstrated a higher mean response rate; however, this difference is not statistically significant (*p* = 0.265). Smoking is known to affect both innate and adaptive immunity, influencing various immune cells including B cells, T helper cells, memory T/B lymphocytes, dendritic cells, macrophages, and natural killer cells [35]. Studies have shown that cigarette smoking not only impairs the immune response to infections but also reduces the immune reaction during extended chronic infection, resulting in cross-reactive immunopathology [35]. Smokers are reported to have a 29% greater likelihood of displaying nonprotective antibody titers compared to nonsmokers [36]. This adverse effect is associated with nicotine's effects on the T cell antigen–mediated pathway and intracellular calcium response, which subsequently limits the response of antibody-forming cells [25, 37].

The effect of alcohol on the immune response to the hepatitis B vaccine varies depending on consumption levels; moderate intake may enhance immune function while reducing inflammation, whereas chronic consumption can lead to an impaired response due to immune dysregulation [38]. Alcohol significantly affects various components of both innate and adaptive immunity—including T cells, B cells, macrophages, and neutrophils—yet the specific mechanisms behind hepatitis B vaccine nonresponse remain unclear [39]. Alcohol-induced suppression of B cell activity may decrease antibody production against HBV antigens, potentially contributing to chronic HBV infection [40]. Additionally, since B cells also act as antigen-presenting cells, their reduced numbers due to alcohol consumption could hinder antigen presentation and further compromise immunity [41]. Chronic alcohol use inhibits B cell responsiveness to cytokines such as IL-2 and IL-4, which are critical for B cell differentiation and antibody production [42].

The negative correlation observed (Table 3) between age and vaccine response (*ρ* = −0.296, *p* < 0.01) aligns with existing studies that report older individuals exhibiting a diminished immune response to vaccinations compared to younger populations. This trend can be attributed to immunosenescence, where the aging immune system exhibits reduced efficacy in responding to vaccines due to changes in T-cell and B-cell functionality [13]. In older adults, immunosenescence leads to a weakened adaptive immune response, resulting in a reduced thymic size, impaired T-cell production and differentiation, and decreased antibody production. Previous studies have shown that older populations frequently exhibit lower antibody titers following vaccinations for various pathogens, including influenza and COVID-19. Additionally, older adults are more likely to have comorbidities that can impair immune function, further complicating their responses to vaccines [43]. Moreover, a decline in CD4 T-cells reduces antibody production as a result of inadequate activation of the germinal center, while an increased loss of CD28 molecules on T-cells contributes to T-cell anergy and apoptosis [44]. As individuals age, there is also a deficit in CD62L, an adhesion molecule essential for facilitating the migration of undifferentiated and memory T-cells to lymph nodes. The reduced expression of CD62L further impairs T-cells activation of B-cells, leading to a reduction in antibody production [45].

The correlation between BMI and vaccine response (*ρ* = −0.131, *p* = 0.071) indicates a weak negative trend, suggesting that higher BMI may result in lower hepatitis B vaccine efficacy, although this result was not statistically significant (Table 3). Previous studies have indicated that obesity can impair immunological responses due to continuous low-grade inflammation associated with excess adipose tissue. This inflammatory milieu leads to increased levels of proinflammatory cytokines such as IL-6 and TNF-α, which can negatively affect immune cell function and hinder the body's ability to mount effective responses to vaccines [45, 46]. Obesity has been linked to impaired T-cell activation and functionality. Chronic inflammation can lead to a decrease in the production of essential effector molecules like granzyme B and IFN-γ, which are critical for an effective immune response [46]. Additionally, studies have shown that CD8+ T cell activation is significantly impaired in individuals with obesity, further compromising their ability to respond effectively to vaccinations [47].

## 5. Study Limitations

This study has some limitations that could influence the interpretation of the results. First, using a convenience sampling technique may have introduced selection bias, potentially affecting the representativeness of the findings across a broader Nigerian population. Second, while self-reported data on medical history and lifestyle factors were collected, there is a potential for recall bias or inaccuracies, especially concerning past infections or lifestyle habits. The small sample size, particularly in certain subgroups (e.g., smokers), may have limited the statistical power to detect significant associations with vaccine response. Additionally, the study did not consider genetic factors, which have been shown to influence vaccine response and may partly explain the observed nonresponse rates. Furthermore, the lack of longitudinal follow-up precludes conclusions about the long-term persistence of immunity post-vaccination, which is relevant given HBV's chronic nature. Future studies should address these limitations by incorporating larger, randomized samples, genetic analyses, and extended follow-up to assess long-term vaccine efficacy and immunity.

## 6. Conclusion

This study confirms a high vaccine response rate (94.3%) in a Nigerian population while identifying older age, male gender, and higher BMI as key factors influencing vaccine nonresponse. These findings highlight the need for specific measures to optimize hepatitis B vaccination strategies. We recommend that anti-HB antibody levels should be routinely tested after the completion of the vaccination schedule to identify individuals who fail to develop protective immunity. For nonresponders (anti-HBs < 10 IU/L), additional doses or booster vaccinations should be administered to enhance antibody production. In particular, at-risk populations—including older adults, males, and individuals with higher BMI—may require modified vaccination approaches, such as higher vaccine doses or an extended immunization schedule to improve response rates. Strengthening vaccination policies, public awareness campaigns, and post-vaccination serological testing will be essential in improving hepatitis B control and reducing transmission in Nigeria. Future research should explore long-term vaccine efficacy and genetic determinants of nonresponse to inform more personalized immunization strategies. Further research is needed on the genetic determinants of vaccine response, particularly in relation to HBV genotype E, the predominant genotype in Nigeria and West Africa. This genotype has limited genetic diversity and high endemicity, yet its potential impact on vaccine efficacy remains underexplored.

## Figures and Tables

**Table 1:** Included participants' demographic information.

Variables	Range	Mean	Std. deviation
Age (years)	20–55	27.21	6.76
Weight (kg)	40–100	59.96	11.75
Height (m)	1.45–1.90	1.68	0.09
BMI (kg/m^2^)	14.0–39.0	20.69	3.91

*Note:* The participants' ages ranged from 20 to 55, with a mean of 27.21 years. Their weights ranged from 40 to 100 kg, with an average of 59.96 kg. Heights ranged from 1.45 m to 1.90 m, with an average of 1.69 m. The participants' BMI ranged from 13.0 to 31.0, with a mean of 20.69 kg/m^2^.

**Table 2:** Comparative analysis of mean response based on demographic and health variables.

Variable	Mean response ± SD (IU/L)	T value	*p* value
*Gender*			
Male (*n* = 116)	203.21 + 137.13	−2.167	0.031
Female (*n* = 76)	245.68 + 125.90		

*History of TB*			
Yes (*n* = 16)	218.81 + 129.43	−0.037	0.970
No (*n* = 176)	220.13 + 134.86		

*History of recurrent malaria*			
Yes (*n* = 43)	203.54 + 135.03	−0.914	0.362
No (*n* = 149)	224.77 + 133.89		

*History of measles*			
Yes (*n* = 22)	214.12 + 136.63	1.703	0.090
No (*n* = 170)	265.59 + 104.14		

*Smoking*			
Yes (*n* = 2)	150.71 + 122.08	−0.734	0.464
No (*n* = 190)	220.75 + 133.63		

*Alcohol*			
Yes (*n* = 6)	280.20 + 45.59	1.118	0.265
No (186)	218.08 + 135.59		

**Table 3:** Result for the analysis of the relationship between age, BMI, and vaccine response.

Variable	Spearman correlation coefficient (*ρ*)	*p* value (2-tailed)
Age vs. vaccine response	−0.296	< 0.01
BMI vs. vaccine response	−0.131	0.071

*Note:* Mean age: 27.2 years; mean BMI: 20.7 kg/m^2^.

## Data Availability

The data that support the findings of this study are available from the corresponding author upon reasonable request.
